# Supplementary figures and images for: Machine learning-based personalized composite score dissects risk and protective factors for cognitive and motor function in older participants
Source: Front Aging Neurosci. 2024 Oct 15;16:1447944. doi: 10.3389/fnagi.2024.1447944 (PMC11518739; doi:10.3389/fnagi.2024.1447944)

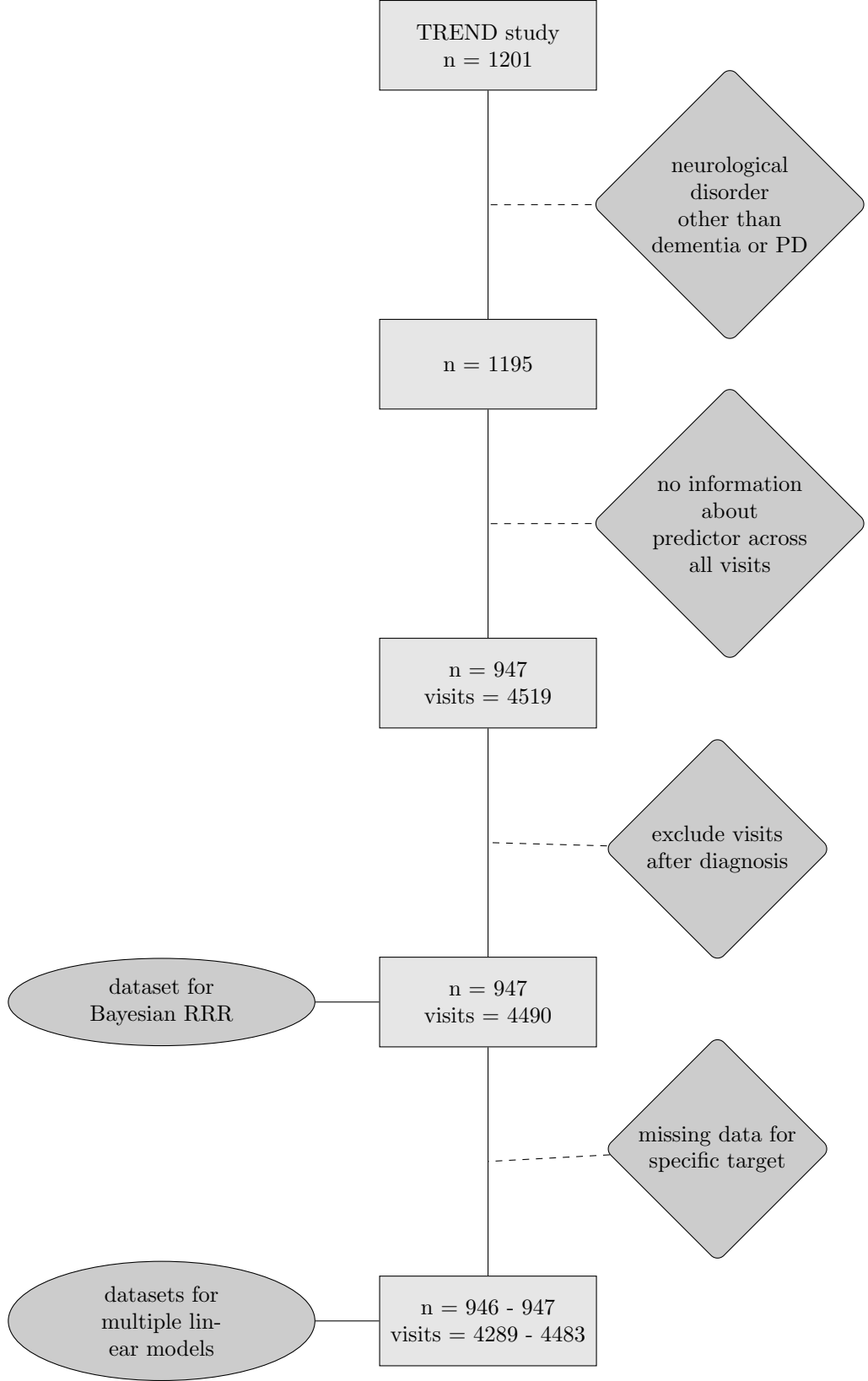

Supplement: Supplementary file 1 [file Image_1.PDF]

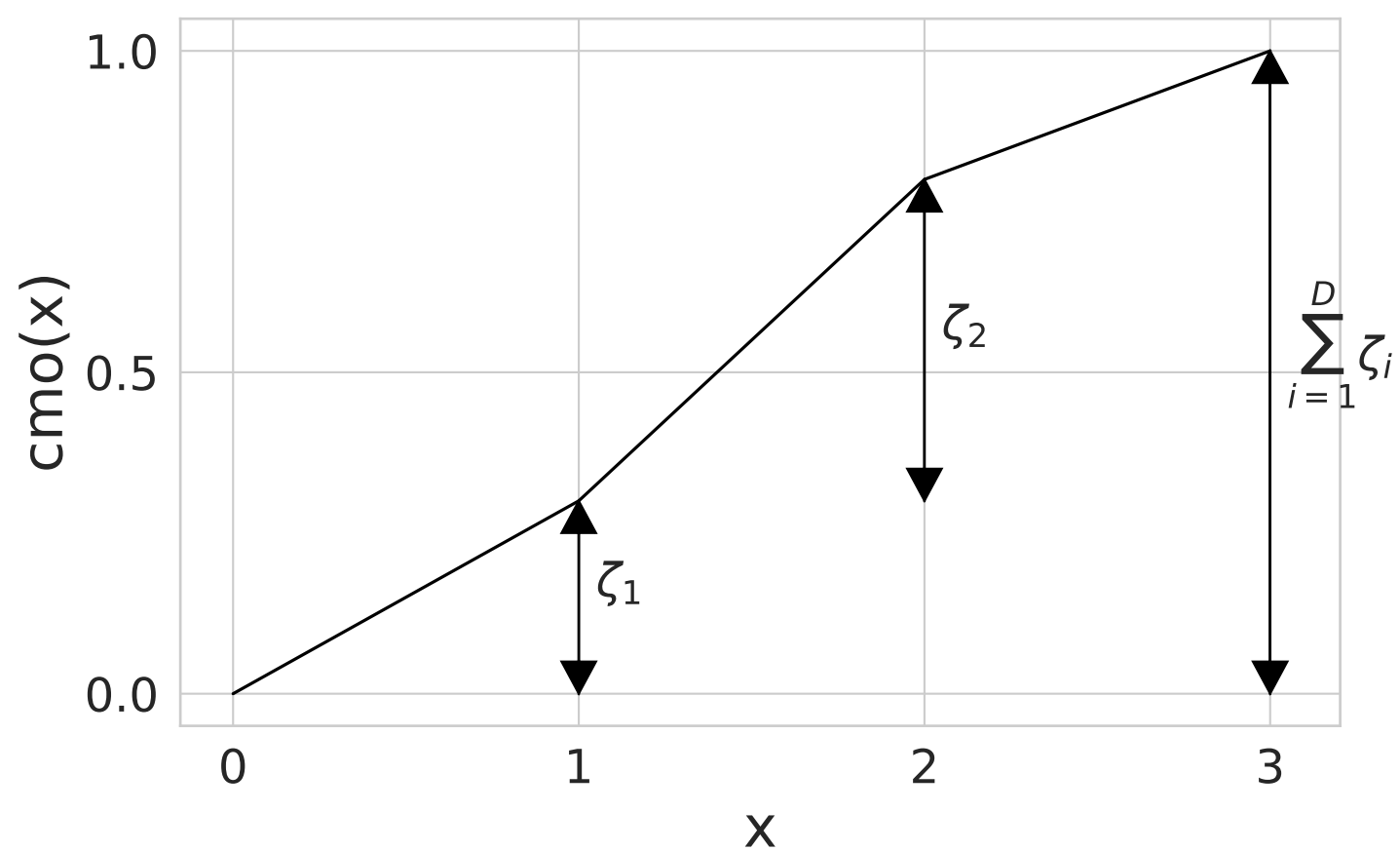

Supplement: Supplementary file 2 [file Image_2.PDF]

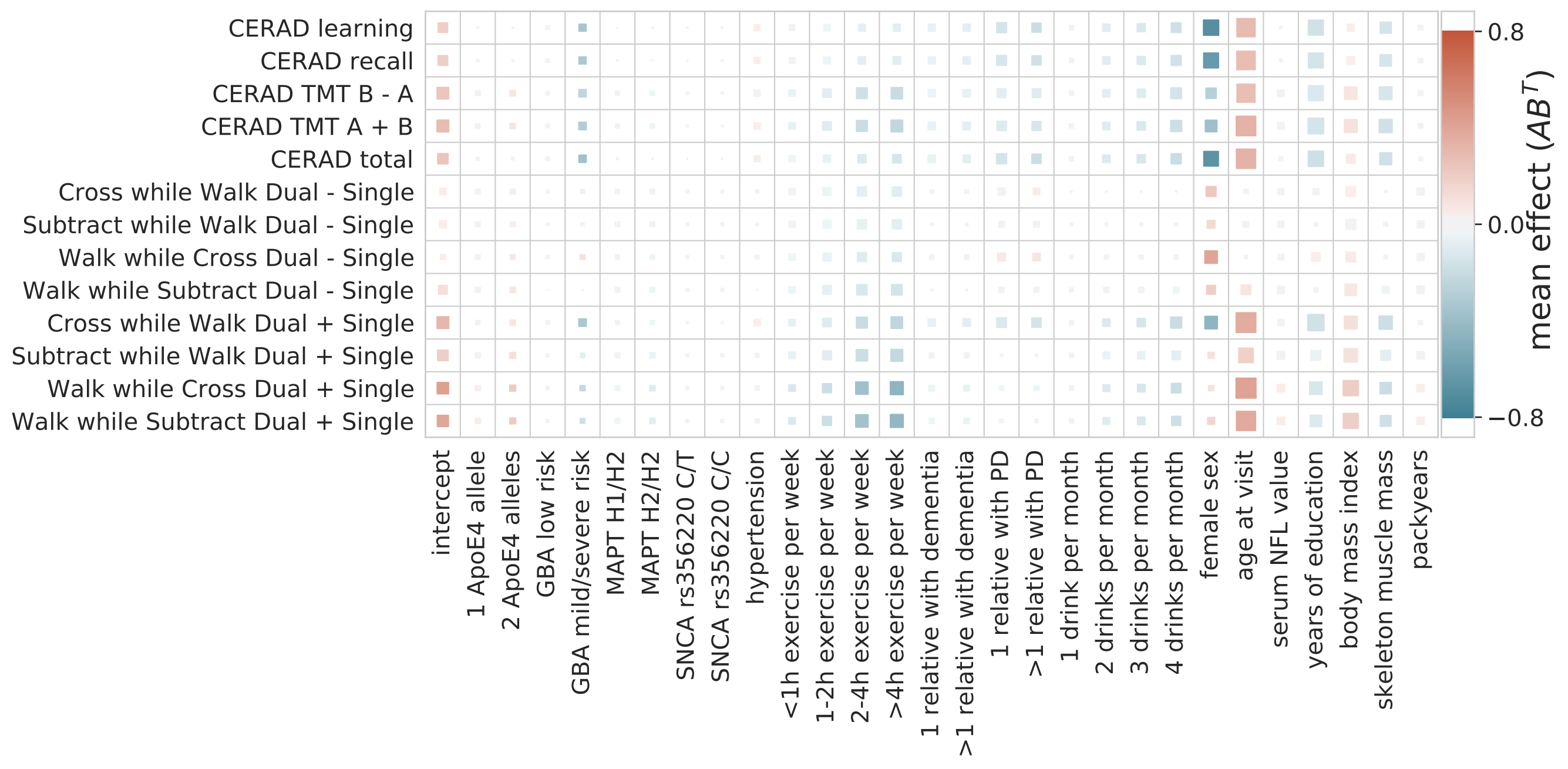

Supplement: Supplementary file 3 [file Image_3.PDF]

score 1: cognitive-like

score 2: gait-like

— risk factor — protective factor — not significant

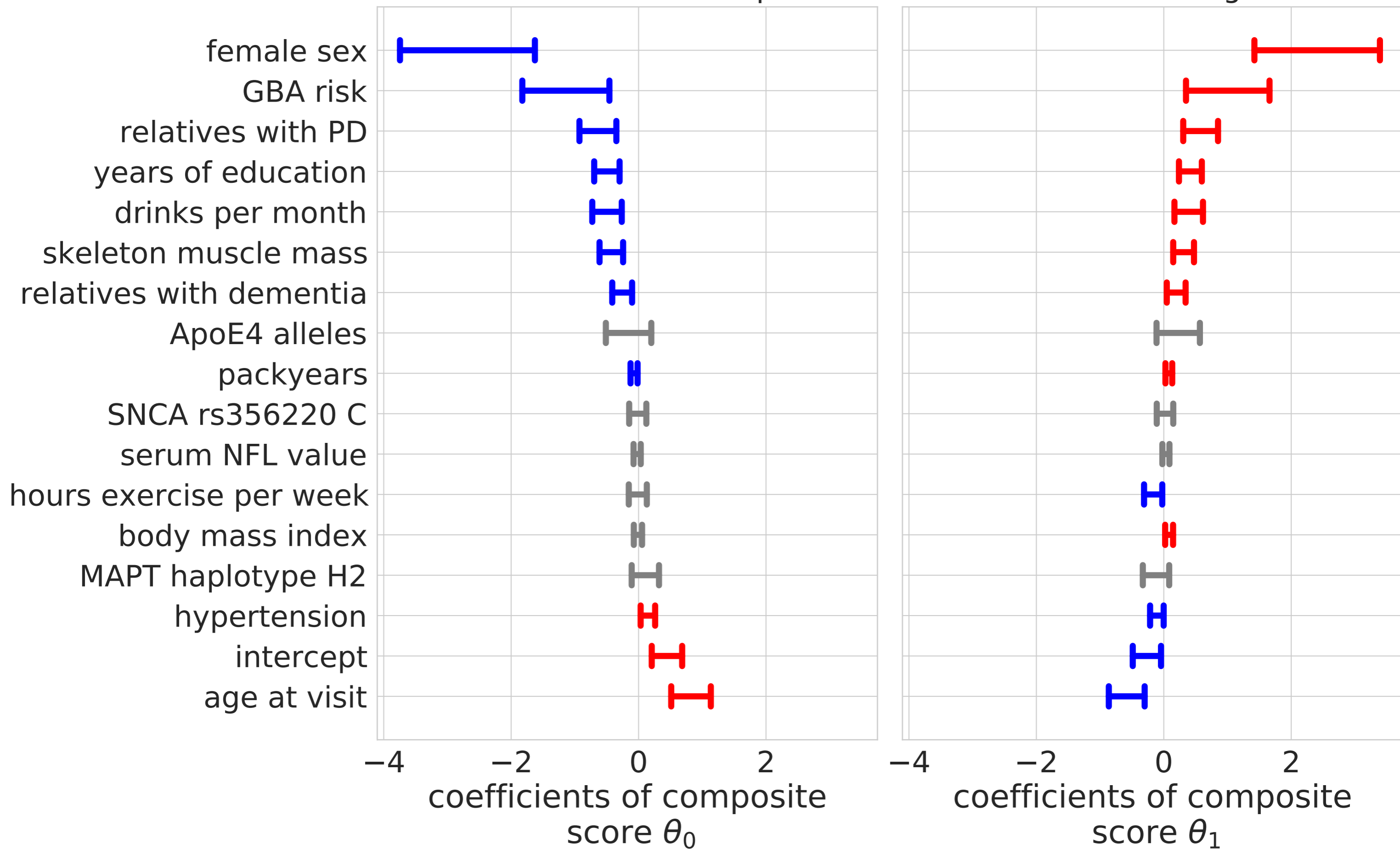

Supplement: Supplementary file 4 [file Image_4.PDF]
