# Supplementary material for: Machine learning-based personalized composite score dissects risk and protective factors for cognitive and motor function in older participants
Source: Front Aging Neurosci. 2024 Oct 15;16:1447944. doi: 10.3389/fnagi.2024.1447944 (PMC11518739; doi:10.3389/fnagi.2024.1447944)
Supplement: Supplementary file 5 [file Table_1.DOCX]

| normal/categorical data | % missing | mean | std |
| --- | --- | --- | --- |
| age at visit | 0 | $66.828$ | $7.131$ |
| serum NFL | $19.065$ | $16.595$ | $16.595$ |
| years of education | 0 | $14.443$ | $2.665$ |
| female sex | 0 | $0.487$ | $0.500$ |
| converts to PD during study | 0 | $0.015$ | $0.122$ |
| converts to dementia during study | 0 | $0.018$ | $0.135$ |
| body mass index | $0.646$ | $26.112$ | $4.224$ |
| skeleton muscle mass | $52.183$ | $26.091$ | $6.678$ |
| packyears | $7.572$ | $6.214$ | $12.232$ |
| hypertension | 0 | $0.611$ | $0.488$ |
| ordinal data | $\%$ missing | median | $[25\%,75\%]$ |
| apoe4 alleles | 0 | 0 | $[0,0]$ |
| tau haplotype H2 | 0 | 0 | $[0,1]$ |
| gba pd risk | 0 | 0 | $[0,0]$ |
| snca rs356220 C | 0 | 1 | $[1,2]$ |
| hours exercise per week | $21.114$ | 3 | $[2,3]$ |
| drinks per month | $33.185$ | 4 | $[2,4]$ |
| relatives with PD | 0 | 0 | $[0,0]$ |
| relatives with dementia | 0 | 0 | $[0,1]$ |
