# Supplementary material for: Machine learning-based personalized composite score dissects risk and protective factors for cognitive and motor function in older participants
Source: Front Aging Neurosci. 2024 Oct 15;16:1447944. doi: 10.3389/fnagi.2024.1447944 (PMC11518739; doi:10.3389/fnagi.2024.1447944)
Supplement: Supplementary file 6 [file Table_2.DOCX]

| ordinal data | count | median | $[25\%,75\%]$ |
| --- | --- | --- | --- |
| CERAD total | 4472 | 14 | $[9,20]$ |
| CERAD learning | 4399 | 9 | $[7,12]$ |
| CERAD recall | 4483 | 3 | $[2,5]$ |
| normal data | count | mean | std |
| CERAD TMT B - A | 4425 | $51.170$ | $34.758$ |
| CERAD TMT A + B | 4425 | $126.188$ | $48.771$ |
| Cross while Walk Dual - Single | 4383 | $-0.279$ | $0.264$ |
| Subtract while Walk Dual - Single | 4289 | $-0.010$ | $0.114$ |
| Walk while Cross Dual - Single | 4399 | $-0.219$ | $0.166$ |
| Walk while Subtract Dual - Single | 4312 | $-0.298$ | $0.207$ |
| Cross while Walk Dual + Single | 4383 | $-2.727$ | $0.521$ |
| Subtract while Walk Dual + Single | 4289 | $-0.679$ | $0.292$ |
| Walk while Cross Dual + Single | 4399 | $-3.037$ | $0.463$ |
| Walk while Subtract Dual + Single | 4312 | $-2.962$ | $0.453$ |
