# Supplementary material for: Machine learning-based personalized composite score dissects risk and protective factors for cognitive and motor function in older participants
Source: Front Aging Neurosci. 2024 Oct 15;16:1447944. doi: 10.3389/fnagi.2024.1447944 (PMC11518739; doi:10.3389/fnagi.2024.1447944)
Supplement: Supplementary file 7 [file Table_3.DOCX]

| k | 1 | | 2 | | single task | |
| --- | --- | --- | --- | --- | --- | --- |
| test | t-statistic | p-value | t-statistic | p-value | t-statistic | p-value |
| CERAD learning | -0.771582 | 0.462542 | 0.903303 | 0.392751 | 1.18038 | 0.271753 |
| CERAD recall | 0.390882 | 0.706088 | 1.447446 | 0.185797 | 1.59984 | 0.148302 |
| CERAD TMT B - A | 0.761247 | 0.468353 | 0.274502 | 0.790655 | 0.726159 | 0.488439 |
| CERAD TMT A + B | 0.579235 | 0.578372 | -0.382270 | 0.712216 | 1.14434 | 0.285564 |
| CERAD total | -0.624379 | 0.549766 | -0.097527 | 0.924707 | 1.2544 | 0.245106 |
| Cross while Walk Dual - Single | -2.136732 | 0.065107 | -0.979398 | 0.356069 | -1.16826 | 0.276338 |
| Subtract while Walk Dual - Single | -2.978478 | 0.017641 | -1.003217 | 0.345132 | -0.740939 | 0.479911 |
| Walk while Cross Dual - Single | -3.184523 | 0.012909 | -0.341609 | 0.741445 | -0.353704 | 0.732701 |
| Walk while Subtract Dual - Single | -0.722037 | 0.490835 | 0.060063 | 0.953579 | -0.193342 | 0.85151 |
| Cross while Walk Dual + Single | -2.050907 | 0.074403 | -2.874170 | 0.020696 | -1.22074 | 0.25694 |
| Subtract while Walk Dual + Single | 0.445314 | 0.667896 | 0.179760 | 0.861811 | 0.584819 | 0.574788 |
| Walk while Cross Dual + Single | -2.717913 | 0.026334 | -4.846756 | 0.001277 | -0.332764 | 0.747865 |
| Walk while Subtract Dual + Single | -2.956444 | 0.018245 | -5.664771 | 0.000473 | -0.198769 | 0.847402 |
